# Maintaining trust in uncertain times: Funding pauses and the ethical cost to community‐engaged research

**DOI:** 10.1002/ajcp.70028

**Published:** 2025-11-14

**Authors:** Brynn E. Sheehan

**Affiliations:** ^1^ Virginia Health Sciences at Old Dominion University Norfolk Virginia USA

**Keywords:** community‐engaged research, trust, funding instability, research ethics, research continuity

## Abstract

Federal funding pauses, once considered rare, are increasingly disrupting the stability and continuity of community‐engaged research. Even projects with active, awarded grants are experiencing the strain of funding uncertainty, raising substantive concerns among research teams and community partners. These disruptions extend beyond operational delays; they jeopardize the continuity of relationships developed through sustained, collaborative engagement. In the absence of clear information about a project's future, uncertainty undermines trust and challenges the ethical principles that guide human subjects research, particularly beneficence and justice. This paper draws on direct experience and insights from the broader field to examine the practical and ethical implications of funding instability in community‐engaged research contexts. It further outlines structural recommendations to mitigate harm, including the establishment of bridge funding mechanisms, the integration of pause‐contingency planning into grant proposals, and enhanced transparency from funding agencies. Institutional review boards and oversight entities may consider developing clear guidance for maintaining ethical obligations during funding disruptions. Research continuity must be recognized not as a procedural convenience, but as a foundational element of ethical research practice. Upholding the core values of community‐engaged research necessitates systems explicitly designed to promote stability, accountability, and sustained partnership, even amid an increasingly unpredictable funding environment.

## Highlights

- Funding pauses disrupt even active grants, eroding trust in community‐engaged research.
- Instability challenges ethical principles, including beneficence and justice.
- Structural solutions are needed to protect research continuity and uphold ethical commitments.

“Is the study still going on?” The question, asked by a community partner during one of our weekly community advisory board (CAB) meetings, was both straightforward and deeply consequential. I answered honestly, with a tempered sense of optimism, because that is what researchers often do; we develop rigorous protocols, submit our strongest work, and proceed with the hope, never the guarantee, that funding will be awarded. Historically, once that funding is secured, researchers operate with an understanding that it will remain stable, at least until the end of the grant period, contingent upon progress. That foundation now feels increasingly uncertain.

A team member who directs a state‐administered program funded by federal dollars, recently experienced this firsthand when their grant, which fully supports their own salary and benefits as well as five others, was thrown into uncertainty just days before the start of a new fiscal year. With no communication from the funder and no clarity regarding timelines, leaders worked urgently to identify bridge funds to support six team members facing potential immediate job loss. Although partial funding was ultimately confirmed only two business days before the new fiscal year began, the disruption had already taken its toll. One team member had accepted a more stable position elsewhere, and the remaining staff were unsettled. Even with the project now operational, at least temporarily, the sense of security that once sustained their work has understandably not been fully restored.

My own research team is navigating similar concerns. Our grants have not been rescinded or formally paused, but the anxiety among staff is genuine. We have convened multiple meetings to discuss potential scenarios, contingency plans, and implications for the communities with which we partner. Even in the absence of a formal pause, the uncertainty is already influencing morale, decision‐making, and long‐term strategy. We are not only competing for new funding; we are now navigating the instability of funding that has already been awarded.

To those outside the research ecosystem, federal funding pauses may seem like routine procedural matters. In practice, however, they create significant disruptions. Teams are unable to hire, critical supplies are delayed, and time‐sensitive project milestones are missed. In community‐engaged research, the consequences are especially severe. Even seemingly minor administrative pauses, such as delays in processing payments, can be magnified for community partners and participants, many of whom face financial precarity. What may to some feel like a short‐term inconvenience within a university system can translate into immediate and destabilizing hardship in community settings.

Our work is not limited to reporting findings or delivering services; we build research collaboratively with communities, grounded in shared decision‐making, mutual accountability, and long‐standing trust (Wallerstein, 2021). These principles are central to community‐based participatory research and the Engage for Equity framework, which emphasizes that institutional and structural support is essential to sustaining equitable partnerships. This commitment also reflects broader calls within community psychology to reengage with its roots in social justice and community mental health (Townley et al., 2018). When we, as researchers, cannot provide clear answers about the continuity of a project, it is not only the research that suffers—it is the relationship. Funding uncertainty erodes these foundational elements, threatening both research outcomes and the ethical fabric of community engagement.

Importantly, these issues are affecting active, funded projects. Conversations with colleagues have surfaced recurring themes: experienced staff departing mid‐project due to instability, clinical studies delaying sample analysis, and early‐career researchers questioning the sustainability of their career paths (Rosenbush, 2025). These are not isolated incidents. They reflect a broader, more systemic disruption that is relatively new. Researchers have always navigated competitive grant environments and lean funding conditions, but there was an expectation that once funding was awarded, it would be issued as planned. That assumption no longer holds. We are working in a reality where even awarded funding can disappear, however temporarily, without warning, and the effects will be long‐lasting.

This form of instability presents not only logistical challenges, but ethical ones as well. For studies involving ongoing interventions, particularly those with clinical or implantable components, the stakes are significantly higher. In some ongoing trials, participants have received implantable devices that require long‐term monitoring and follow‐up care. If funding becomes unavailable to support those obligations, investigators are left with untenable options: continue without sufficient resources, potentially compromising participant safety, or terminate the study prematurely, which could violate IRB‐approved protocols. As a member of an institutional review board, I have witnessed the strain that occurs when research aims outlast financial support. Our regulatory infrastructure assumes continuity of care. But what happens when funding abruptly disappears? These scenarios directly implicate the ethical principles of beneficence and justice, as outlined in the Belmont Report (National Commission for the Protection of Human Subjects of Biomedical and Behavioral Research, 1979). When research teams are unable to fulfill follow‐up obligations due to funding pauses, the risk of harm to participants increases, undermining beneficence. Moreover, the burden of that harm often falls disproportionately on communities that have invested time, trust, and partnership into the research process, raising critical concerns about justice. These are not abstract principles; they are lived realities for participants and communities when the system fails to uphold its commitments.

The impact of this uncertainty extends beyond research teams and regulatory bodies—it reaches community partners. In community‐engaged and community‐based participatory research, our collaborators are not passive recipients of services or data; they are active co‐creators of research, whose time, labor, and trust are invested in the work. One way to support this investment is through structural governance mechanisms such as CABs and co‐leadership models, which embed shared decision‐making and accountability into projects (Sanchez‐Youngman et al., 2021). CABs, once primarily used as an ethical approval mechanism, now play a broader role in guiding research priorities, improving study quality, and ensuring projects remain responsive to community needs (Newman et al., 2011). When meaningfully engaged, such governance structures not only strengthen research alignment with community priorities but may also provide a mechanism for communities to steward projects through periods of disruption, including funding pauses. However, when community engagement is undervalued or inconsistently supported, inequities deepen, and the ethical foundation of participatory research is weakened (Rodriguez Espinosa & Verney, 2021). Increasingly, our CAB members are asking questions such as, “What is going to happen with this project?” and “Is it even worth applying for the next grant?” These questions emerge not from disengagement, but from deep investment in the research process. The trust required to build and sustain these partnerships takes years to establish. My colleagues, research team members, and I are committed to maintaining these relationships, regardless of funding status. However, the growing inability to offer clear, confident answers, through no fault of our own, risks eroding that trust. If partnerships begin to fray due to systemic instability, the consequences will be far‐reaching and difficult to repair.

What we as researchers, particularly community‐engaged researchers, are experiencing is not merely a fiscal challenge. It is a disruption to the ethical and professional contract that researchers, funders, and communities have long upheld. These are undeniably uncertain and unstable times for universities and institutions as many are navigating their own financial pressures, staffing shortages, and administrative delays. However, that reality does not absolve us of the responsibility to plan for the future. On the contrary, it heightens the need for intentional, proactive strategies to mitigate harm when disruptions arise. As a community of researchers, universities, and funders who support the research, we must establish new mechanisms to support continuity during periods of federal funding uncertainty.

## RECOMMENDATIONS

Table 1 outlines recommended actions to anticipate and respond to funding pauses in community‐engaged research, mapped to the ethical principles of beneficence, justice, and respect for persons, and the entities responsible for their implementation. Funding agencies and institutions should consider dedicated bridge funding, operational reserves, or policy flexibilities that allow for minimal study maintenance during pauses. While dedicated bridge funding from universities can provide vital continuity during funding interruptions, few institutions have mechanisms sufficient to sustain community‐engaged or longitudinal studies. Nevertheless, this commentary calls for institutions to prioritize and reorganize funding structures to better safeguard this type of work. In some cases, philanthropic or industry partnerships may help fill these gaps; however, these sources present additional ethical and equity considerations. Philanthropic funding often reflects donor priorities, which may not align with community needs, and these entities are not unlimited. Many have already experienced sharp increases in funding requests amid federal cuts and pauses. Similarly, industry support can raise concerns about conflicts of interest or influence on research agendas. Transparent governance and clear ethical guidelines are therefore essential to ensure that stopgap solutions do not inadvertently compromise the integrity or independence of community‐engaged research. Additionally, funders could support automatic grace periods or short‐term no‐cost extensions to prevent critical disruptions, particularly when delays stem from administrative factors beyond investigators’ control, such as federal shutdowns or agency processing delays. At the time of grant applications, investigators could be encouraged to submit ‘pause plans’ describing how studies would ethically continue under short‐term funding gaps. Institutions might also explore cross‐project resource sharing, such as collaborative models that enable departments to share personnel, equipment, and infrastructure, as well as emergency continuity funds, or contingency staffing approaches that allow teams to shift roles temporarily rather than disband entirely.

**Table 1:** Recommended actions to mitigate the impact of funding pauses in community‐engaged research, the ethical alignment, and responsible entities.

	Suggestion	Ethical Principle(s)	Primary Entity Responsible
1.	Dedicated bridge funding	Justice	Funder, Institution
2.	Operational reserves	Justice	Institution, Researcher
3.	Policy flexibilities for minimal study maintenance	Beneficence	Funder, Institution
4.	Automatic grace periods or short‐term no‐cost extensions	Beneficence and Justice	Funder
5.	Pause plans submitted during original application, to describe how the study will ethically continue under short‐term funding gaps	Beneficence and Respect for Persons	Funder, Researcher
6.	Cross‐project resource sharing	Justice	Institution, Researcher
7.	IRB‐related guidance during funding pauses, particularly concerning participant safety	Beneficence	IRB
8.	Expedited approval of protocol modifications	Beneficence and Respect for Persons	IRB
9.	Funding updates and processing timelines	Respect for Persons	Funder
10.	Consistent communication with community partners	Justice and Respect for Persons	Researcher, Community Partner
11.	Inclusion of community advisory boards in contingency plans	Justice and Respect for Persons	Researcher, Community Partner

Institutional review boards and oversight bodies may also need to develop clearer guidance for navigating studies caught in limbo, particularly when participant safety and ethical obligations are at stake. This could include defining standards for ethical minimal maintenance and allowing expedited approval of protocol modifications during funding interruptions. Transparency is also critical; funders should provide real‐time updates and processing timelines, while researchers take proactive steps to keep participants and community partners informed. CABs should be included in contingency planning, not as an afterthought but as co‐creators of responsible, supportive solutions. Without such safeguards, we risk normalizing instability and weakening the ethical foundation of our work.

## CONCLUSIONS

If we are to uphold the ethical obligations of research, that is, beneficence, justice, and respect for persons, then we must also uphold the conditions that make these obligations possible. Research continuity is not a bureaucratic detail; it is a cornerstone of ethical research practice. It ensures that investigators can honor their commitments, that communities are not left behind, and that public trust in science has a chance to be preserved. Hope cannot be the only resource that sustains a project. Researchers and the community partners who help implement and sustain projects deserve more than reassurances; they deserve systems built for stability, transparency, and accountability. Until continuity is treated as essential to research ethics, the integrity of projects and the very relationships that sustain meaningful, community‐centered science, will continue to be jeopardized.

## CONFLICT OF INTEREST STATEMENT

The author declares no conflicts of interest.

## Data Availability

Data sharing is not applicable to this article, as no datasets were generated or analyzed during the current study.
